# Role of Fibronectin in the Adhesion of *Acinetobacter baumannii* to Host Cells

**DOI:** 10.1371/journal.pone.0033073

**Published:** 2012-04-13

**Authors:** Younes Smani, Michael J. McConnell, Jerónimo Pachón

**Affiliations:** Clinic Unit of Infectious Diseases, Microbiology and Preventive Medicine, Institute of Biomedicine of Seville, IBiS, University Hospital Virgen del Rocío/CSIC/University of Seville, Seville, Spain; University of Pittsburgh, United States of America

## Abstract

Adhesion to host cells is an initial and important step in *Acinetobacter baumannii* pathogenesis. However, there is relatively little information on the mechanisms by which *A. baumannii* binds to and interacts with host cells. Adherence to extracellular matrix proteins, such as fibronectin, affords pathogens with a mechanism to invade epithelial cells. Here, we found that *A. baumannii* adheres more avidly to immobilized fibronectin than to control protein. Free fibronectin used as a competitor resulted in dose-dependent decreased binding of *A. baumannii* to fibronectin. Three outer membrane preparations (OMPs) were identified as fibronectin binding proteins (FBPs): OMPA, TonB-dependent copper receptor, and 34 kDa OMP. Moreover, we demonstrated that fibronectin inhibition and neutralization by specific antibody prevented significantly the adhesion of *A. baumannii* to human lung epithelial cells (A549 cells). Similarly, *A. baumannii* OMPA neutralization by specific antibody decreased significantly the adhesion of *A. baumannii* to A549 cells. These data indicate that FBPs are key adhesins that mediate binding of *A. baumannii* to human lung epithelial cells through interaction with fibronectin on the surface of these host cells.

## Introduction

The ability of bacteria to interact with eukaryotic cells, leading to their own internalization, seems to be a critical event in the pathogenesis process of several microorganisms [1]. Invasive bacteria reach a compartment in which they are protected against host clearance mechanisms, can replicate and prepare themselves to gain access to tissues and circulatory system.

*Acinetobacter baumannii* have long been considered as nosocomial pathogen with low virulence. However, several recent studies have shown that this microorganism is more virulent than expected [2]. Interaction between *A. baumannii* and the host epithelial cells is important in determining the outcome of infections. Various studies have shown that *A. baumannii* are able to adhere to and invade human epithelial cells; and induce epithelial cells death [3]–[6]. However, there is relatively little information on the mechanisms by which *A. baumannii* bind to and interact with host cells.

Since the initial reports on *A. baumannii* adherence and invasion, attempts have been made to elucidate the mechanisms by which *A. baumannii* promote adherence and invasion in host cells. *Acinetobacter sp* and *A. baumannii* used pili, fimbrial-like structures and outer membrane protein A (OmpA) to facilitate its adhesion and invasion in host cells [3], [5], [7]. However, the host cells surface factors that mediate adherence of *A. baumannii* are largely uncharacterized. Potential host cell receptors for *A. baumannii* adhesion can include extracellular matrix (ECM) proteins, such as integrin and fibronectin; which have been used as bridging molecules to achieve the attachment and the invasion of host cells by pathogens like *Pseudomonas aeruginosa*, *Escherichia coli*, *Streptoccocus pyogenes* and *Staphylococcus aureus* [8]–[11].

In support of this, bacterial fibronectin-binding proteins (FBPs) were consistently identified as key players in the process of host cells adhesion to and internalization of different pathogens [12]. FBPs also mediate the attachment of bacteria to implanted material, which becomes coated with host proteins [13], indicating a possible role in infections associated with catheters and prosthetic devices. Most of them are members of large gram negative bacteria, and more thoroughly described in *E. coli* with most extensive attention [12]. At least 10 different proteins from *E. coli* bind to fibronectin leading to internalization of *E. coli* by human host cells including epithelial cells [12].

In the case of *A. baumannii*, only one study has reported *in vitro* that *A. baumannii* adhesive property involves fibronectin [14]. However, the characterization of the role played by this protein is still limited and the FBPs mediating the binding between fibronectin and *A. baumannii* need to be determined. The present study, therefore, aimed to examinate the binding of fibronectin to *A. baumannii* and the identification of the FBPs involved in this process.

## Results

### Interaction of *A. baumannii* with immobilized fibronectin

**Figure 1:**
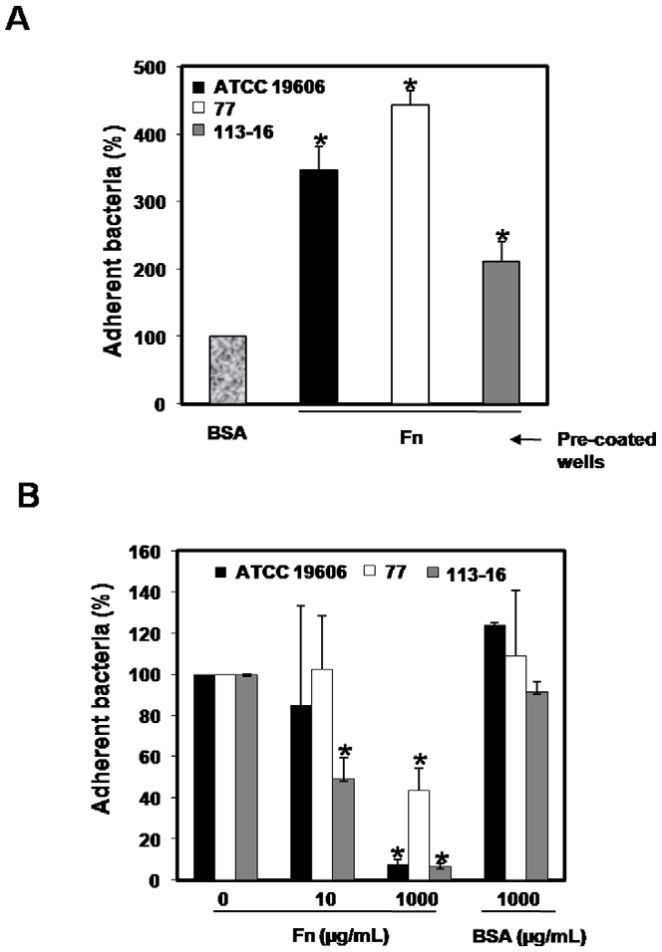
Interaction of *A. baumannii* with immobilized fibronectin. (**A**) *A. baumannii* binding to fibronectin. ATCC 19606, 77 or 113-16 strains were incubated in BSA or in fibronectin-coated wells for 3 h at room temperature. Adherent bacteria were quantified by serial dilutions as described in materials and methods. (**B**) Inhibition of *A. baumannii* adherence to immobilized fibronectin by free fibronectin. ATCC 19606, 77 or 113-16 strain were incubated in fibronectin-coated wells containing increasing concentrations of free fibronectin (0, 10, 100 and 1,000 µg/mL) or BSA (1,000 µg/mL). Adherent bacteria were quantified by serial dilutions as described in materials and methods. Results were expressed as the percentage of total untreated *A. baumannii* adhered to immobilized fibronectin. Representative results of three independent experiments are shown and data are the means ± SEM. *P*<0.05: * between untreated and treated groups. Fn: fibronectin and BSA: bovine serum albumin.

We showed that all *A. baumannii* strains studied here adhered more to fibronectin pre-coated wells than to BSA precoated wells. The adhesion of *A. baumannii* to immobilized fibronectin was significantly greater for 77 and ATCC 19606 strains than for 113-16 strain (Fig. 1A). Moreover, soluble fibronectin (from 10 to 1,000 µg/mL) used as a competitor was able to almost completely inhibit all three *A. baumannii* strains binding with an elective plasmatic fibronectin concentration inhibiting bacterial adherence at 50% (IC_50_) of ≈200, 500 and 10 µg/mL, respectively (Fig. 1B). In contrast, incubation of all three *A. baumannii* strains with BSA (1,000 µg/mL) did not inhibit significantly the binding of *A. baumannii* ATCC 19606, 77 and 113-16 strains to immobilized fibronectin. From these data, we suggest that *A. baumannii* has specific ligands for fibronectin.

### Identification of *A. baumannii* FBPs

**Figure 2:**
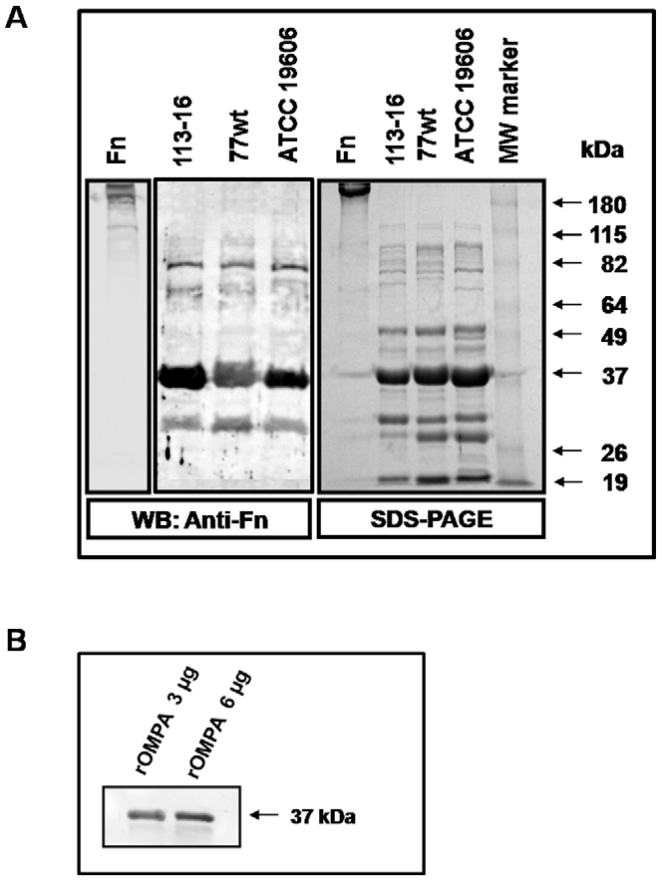
Immunodetection of binding of fibronectin to outer membrane proteins of *A. baumannii* and rOMPA. Immunodetection of FBPs outer membrane of *A.baumannii* ATCC 19606, 77 or 113-16 strain (**A**) and FBP rOMPA (**B**). OMPs were extracted from outer membrane and rOMPA was produced in *E. coli* as described in materials and methods and stained with SimplyBlue™ SafeStain (SDS-PAGE) or electrotransferred onto nitrocellulose membrane, and incubated with Fn. FBPs were probed with rabbit anti- human fibronectin and HRP-conjugated goat anti-rabbit IgG (WB). Molecular mass standards (kDa) are shown on the right. FBPs: fibronectin binding proteins, Fn: fibronectin, MW: molecular weight, WB: western blot.

After growing the 3 strains of *A. baumannii* for 4 h at 37°C, the enriched OMPs electrophoretic profiles were compared. We observed a significant loss of a 29 kDa OMP in the 113-16 strain when compared with the ATCC 19606 and 77 strains. Western blotting analysis performed following 10% SDS-PAGE of OMPs of each *A. baumannii* strain (ATCC 19606, 77 and 113-16) and their transfer to nitrocellulose membranes revealed that incubation of this nitrocellulose membrane with fibronectin (10 µg/mL, 1 h) formed three stables complexes with fibronectin for each strain (Fig. 2A). The three bands have apparent molecular masses of 80, 36 and 32 kDa (Fig. 2A). To identify these proteins which bind the fibronectin, we excised from SDS-PAGE gel the bands representing fibronectin-binding proteins (FBPs) and subjected them to MS-MS/MS analysis. Data obtained from peptide mass fingerprinting were matched against the NCBI database (http://www.ncbi.nlm.nih.gov). The amino acid sequences identification revealed amino acid identity of 48, 51 and 60% with *A. baumannii* TonB-dependent copper receptor, *A. baumannii* OMPA and *A. baumannii* 34 kDa OMP, respectively (Table 1, Data S1, S2, and S3). For each protein, Mascot probability based mowse score with protein score greater than 84 are significant (p<0.05). To confirm the binding of OMPA to fibronectin, we performed the same experiments with the recombinant OMPA (rOMPA) produced by *Escherichia coli*. Western blot analysis showed that rOMPA forms a stable complex with fibronectin (Fig. 2B).

**Table 1:** Identification of FBPs from *A. baumannii* ATCC 19606 by MALDI-TOF-TOF (MS-MS/MS) of selected bands.

Band (kDa)	Sequence coverage (%)	Peptide matched	ID	Protein
78.03	48	See Data S1	gi: 260557574	TonB-dependent copper receptor
36.94	51	See Data S2	gi: 129307154	Outer membrane protein A
32.09	60	See Data S3	gi: 184159810	34 kDa outer membrane protein

### Involvement of fibronectin in the adherence of *A. baumannii* to human lung epithelial cells

**Figure 3:**
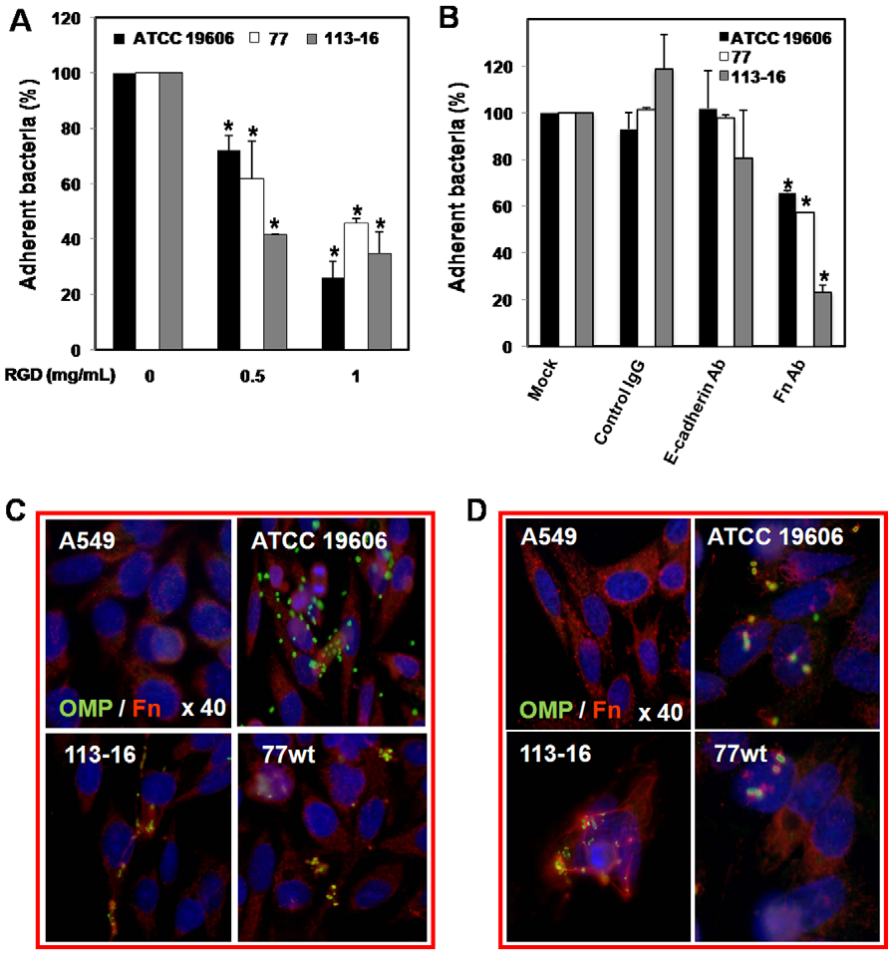
Involvement of fibronectin in the adherence of *A. baumannii* to human lung epithelial cells. A549 cells were pretreated with RGD (0.5 and 5 mg/mL) (**A**), rabbit anti-human fibronectin (1∶25), rat anti-human E-cadherin (1∶25) or control mouse IgG (1∶25) (**B**) and infected with 10^8^ cfu/mL of *A. baumannii* ATCC 19606, 77 or 113-16 strain for 2 h. Adherence assay was performed as described in materials and methods. The effect of treatment on *A. baumannii* adherence to A549 cells is expressed as the percentage of total untreated *A. baumannii* adhered to A549 cells. Immunostaining for A549 cells fibronectin and *A. baumannii* OMPs in infected A549 cells with 10^8^ cfu/mL *A. baumannii* ATCC 19606, 77 or 113 strains for 2 h (**C**) and 24 h (**D**) were performed and imaged by immunofluorescence microscopy. Fibronectin of A549 cells and OMPs of *A. baumannii* strains were detected by rabbit anti-human fibronectin and mouse anti-*A. baumannii* OMPs antibodies and labeled with Alexa594 and Alexa488-tagged the secondary antibodies that appeared red and green, respectively. Blue staining shows the location of A549 cells nucleus. Representative results of three independent experiments are shown and data are the means ± SEM. *P*<0.05: * between untreated and treated groups. Fn: fibronectin and Ab: antibody.

To evaluate the involvement of fibronectin in the *A. baumannii* adherence to A549 cells, we studied the effect of fibronectin inhibitor, RGD, and anti-human fibronectin antibody on *A. baumannii* ATCC 19606, 77 or 113-16 strain adherence to A549 cells for 2 h. We showed that pretreatment of A549 cells with 0.5 and 1 mg/mL of RGD reduced significantly the adherence of ATCC 19606, 77 and 113-16 strains to A549 cells to 72.05±5.39% and 26±6%, 61.74±13.59% and 45.88±1.43%, and 41.49±0.11% and 34.74±7.68%, respectively (Fig. 3A). Pretreatment of A549 cells with RGD did not affect the A549 cells viability (data not shown). Similarly, pretreatment of A549 cells with 28 µg/mL (1∶25) anti-human fibronectin antibody reduced significantly the adherence of ATCC 19606, 77 and 113-16 strains to A549 cells to 65.97±0.69%, 57.33±0.29%, and 23.33±2.93%, respectively. In contrast, pretreatment of A549 cells with unspecific antibody to fibronectin, a mouse IgG, or with rat anti-human E-cadherin antibody, antibody against an irrelevant epithelial surface protein cells, did not reduce significantly the adherence of ATCC 19606, 77 and 113-16 strains to A549 cells (Fig. 3B). The results obtained above with adherence assays were confirmed by fluorescence microscopy experiments, with differential fluorescence labeling used to distinguish between *A. baumannii* and fibronectin of A549 cells. After 2 h incubation of A549 cells with *A. baumannii* ATCC 19606, 77 or 113-16 strain, bacterial cells (green fluorescence) were colocalized with the fibronectin (red fluorescence) in the cell membrane, indicating that they were attached to A549 cells (Fig. 3C). Interestingly, the interaction between *A. baumannii* and fibronectin was more evident after 24 h of A549 cells incubation with *A. baumannii* strains. We observed that bacterial cells (green fluorescence) were colocalized with the fibronectin (red fluorescence) in the cell membrane and inside the cell (Fig. 3D). Altogether, we found that fibronectin plays an important role in the interaction between *A. baumannii* and human lung epithelial cells.

### Involvement of *A. baumannii* OMPA in the adherence of *A. baumannii* to human lung epithelial cells

**Figure 4:**
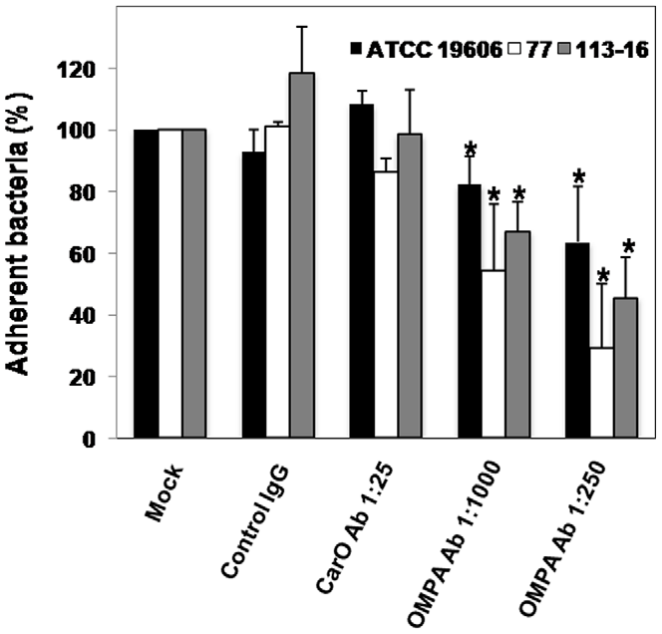
Involvement of *A. baumannii* OMPA in the adherence of *A. baumannii* to human lung epithelial cells. A549 cells were infected for 2 h with 10^8^ cfu/mL of *A. baumannii* ATCC 19606, 77 or 113-16 strain previously incubated for 1 h with mouse anti-OMPA of *A. baumannii* (1∶1,000 and 1∶250), rabbit anti-CarO of *A. baumannii* (1∶25) and control mouse IgG (1∶25). Adherence assay was performed as described in materials and methods. The effect of treatment on *A. baumannii* adherence to A549 cells is expressed as the percentage of total untreated *A. baumannii* adhered to A549 cells. Representative results of three independent experiments are shown and data are the means ± SEM. *P*<0.05: * between untreated and treated groups. OMPA: outer membrane protein A and Ab: antibody.

To demonstrate the involvement of OMPA, a FBP, in the *A. baumannii* adherence to A549 cells, we studied the effect of mouse anti-*A. baumannii* OMPA antibody on *A. baumannii* ATCC 19606, 77 or 113-16 strain adherence to A549 cells for 2 h. We showed that pretreatment of A549 cells with OMPA antibody diluted at 1∶1000 and 1∶250 reduced significantly the adherence of ATCC 19606, 77 and 113-16 strains to A549 cells to 82.46±8.77% and 63.64±18.18%, 54.47±21.56% and 29.17±20.83%, and 67.04±9.71% and 45.65±13.04%, respectively (Fig. 4). In contrast, pretreatment of *A. baumannii* strains with unspecific antibody to fibronectin, a mouse IgG, or with rabbit anti-CarO of *A. baumannii* antibody antibody against CarO which is an outer membrane protein involved in the carbapenem permeability, did not reduce significantly the adherence of ATCC 19606, 77 and 113-16 strains to A549 cells (Fig. 4). From these data, we found that OMPA which is a FBP mediated the interaction between *A. baumannii* and human lung epithelial cells.

## Discussion

*A. baumannii* is an opportunistic pathogen that has emerged as a problematic organism, causing severe infections in human hosts. This is compounded by the fact that clinical isolates of this organism are often resistant to multiple types of antimicrobial therapies. To better understand the mechanisms involved in pathogenicity with the long-term goal of finding targets for novel therapies of the future, the work presented in this manuscript focuses on the factor involved in the interaction of *A. baumannii* to host cells.

A key virulence strategy of Gram-negative pathogens is their ability to interact with proteins of the human serum or ECM. These interactions are associated with bacterial immune evasion or adherence to and invasion into host cells [15]–[17]. In this study, we demonstrated that *A. baumannii* present specific ligands for fibronectin confirming that fibronectin is a potential host for *A. baumannii*. The fibronectin binding represents a preferential receptor for different bacteria [18], [19]. In addition, we showed that *A. baumannii* binding to immobilized fibronectin is mediated by the OMPs. The blocking of the immobilized fibronectin using OMPs extracts, abolished attachment of *A. baumannii* to immobilized fibronectin (data not shown). In agreement with this data, previous studies demonstrate the role played by OMPs in the bacterial binding to fibronectin [20]–[23]. Hence, *A. baumannii* seems to possess the FBPs among their OMPs.

The identification of three FBPs, OMPA, TonB-dependent copper receptor, and 34 kDa OMP, highlights the role of these proteins in the interaction between *A. baumannii* and host cells. The expression of these three FBPs was not affected by the acquired resistance by *A. baumannii*. Although there is no experimental evidence up to now, the interaction and the invasion of *A. baumannii* contribute towards not only systemic infection but also protect the bacteria from antibiotics leading their persistence in the tissue [24], [25].

Interestingly, we identified TonB-dependent copper receptor as a FBP. TonB protein works as an energy transducer coupling cytoplasmic membrane proton motive force to active transport of iron-siderophores and vitamin B12 across the outer membrane of *E. coli* and presumably of other gram-negative bacteria. The function of TonB is apparently to transduce energy to the outer membrane receptors, perhaps by releasing otherwise tightly bound ligands into the periplasmic space [26], [27]. Apart from its capacity to transport molecules, in *Pasteurella multocida* and *Bacteroides fragilis*, TonB is able to recognize and bind to fibronectin [22], [28]. It also has been suggested that TonB may allow some species to adhere to and colonize different niches in the host [28]. Considering that *A. baumannii* OMPA is involved in the interaction between *A. baumannii* and human epithelial cells, in its translocation to mitochondria and cell nucleus, and in its induction of the human epithelial cell death [29]–[32], it is likely that OMPA binding to fibronectin play an important role in this phenomena. In agreement with our data, *P. multocida* have used OMPA to bind to host cells and to interact with fibronectin [33]. In the case of the 34 kDa OMP, we suggest that this FBP is the same protein previously described by different authors as being from 31 to 36 kDa or 33 to 36 kDa [34]–[36]. Similarly to OMPA, it was reported that OMP 33–36 kDa induces the cell death of human cervical epithelial cells (unpublished data). We suggest that this cell death induced by OMP 33–36 kDa would be a consequence of the interaction between OMP 33–36 kDa and host cells.

Adherence-inhibition assays conducted in the presence of fibronectin inhibitor or anti-human fibronectin antibody suggests that host fibronectin may be a specific receptor for *A. baumannii*. In fiboronectin adherence-inhibition assays, the presence of endogenous fibronectin on the surface of human lung epithelial cells may have facilitated the adherence of *A. baumannii* strains to human lung epithelial cells. Presumably, pretreatment with fibronectin inhibitor resulted in its binding to fibronectin on the surface of A549 cells, thus preventing interaction between FBPs and the endogenous cellular fibronectin on the surface of A549 cells. Inhibition resulting from pretreatment of A549 cells with rabbit anti-human fibronectin was more significant for 113-16 strain than ATCC 19606 and 77 strains suggesting the complexity of adherence for susceptible strains and the involvement of multiple factors including other ECM molecules in the bacterium adherence to host cells [33]. Similar results were reported for *P. multocida* [28].

Several studies have shown bacterial adherence to host fibronectin [20], [37], [38]. Bacteria, through their OMPs bind to fibronectin [12]. Our data clearly show that OMPA bind to fibronectin (Fig. 3). Pretreatment of *A. baumannii* with mouse anti-*A. baumannii* OMPA prior incubation with A549 cells strongly reduced the adherence of *A. baumannii* to A549 cells. Similar results have been obtained with *P. multocida* OMPA [33].

In summary, all data reported here indicate that *A. baumannii* binding to fibronectin was mediated by the OMPs. The OMPs were identified as FBPs and were involved in the interaction between *A. baumannii* and fibronectin. Our results encourage us to continue our studies in an attempt to understand the involvement of *A. baumannii* adherence to host cells in the pathogenesis of this pathogen.

## Materials and Methods

### Bacterial strains

*A. baumannii* ATCC 19606 (LGC Standards, United Kingdom) and clinical *A. baumannii* isolate 77, both susceptible to all antimicrobials, and clinical pandrug-resistant *A. baumannii* isolate 113-16 were used. All strains were grown in a Mueller Hinton Broth (MHB) at 37°C for 20–24 h.

### Human cell culture and infection

Type II pneumocyte cell line A549 derived from a human lung carcinoma (LGC Standards, United Kingdom) were grown in DMEM medium supplemented with 10% heat-inactivated foetal bovine serum (FBS), vancomycin (50 µg/mL), gentamicin (20 µg/mL), amphoterecin B (0.25 µg/mL) (Invitrogen, Spain) and 1% HEPES (Gibco, Spain) in a humidified incubator, 5% CO_2_ at 37°C. A549 cells were routinely passaged every 3–4 days. The cells were seeded 24 h in 24 well plates prior to infection with *A. baumannii* strains at a multiplicity of infection (MOI) of 100. Immediately before infection, A549 cells were washed three times with prewarmed phosphate-buffered saline (PBS) and further incubated in DMEM without FBS and antibiotics.

### Coating fibronectin on wells

Coating fibronectin on wells were performed in sterile 96 well plates as described previously [23]. Briefly, wells were coated overnight at 4°C with 1.25 µg of plasmatic fibronectin in PBS (125 µL of fibronectin at 10 µg/mL per well) or with 20 µg/mL of BSA. Then wells were washed four times with 125 µL of 1% (w/v) BSA in PBS, and blocked for 1 h at room temperature with 125 µL of 1% BSA in PBS. Just before adding bacteria, wells were washed six times with PBS.

### Outer membrane preparations and rOMPA production

Outer membrane fraction from *A. baumannii* ATCC 19606, 77 or 113-16 strain were isolated as described previously [39]. Briefly, 1 L of MHB was inoculated with 5 mL of an overnight culture. Bacterial cells were pelleted by centrifugation at 5,000× g for 10 min, resuspended in 10 mL of 10 mM phosphate buffer, pH 7.2, and lysed on ice by sonication for a total of 5–7 min in 30 s intervals. Unlysed cells were pelleted by centrifugation at 5,000× g for 5 min, and the supernatant was centrifuged at 20,000× g for 45 min at 4°C to pellet cell envelopes. Inner membranes from the resulting pellet were solubilized for 30 min at room temperature with 5 mL of 2% N-laurylsarcosinate in 10 mM phosphate buffer, pH 7.2. After solubilization, the insoluble outer membrane fraction was pelleted by centrifugation at 20,000× g for 45 min at 4°C. The outer membrane fraction was washed by resuspension in 2 mL 62.5 mM Tris-HCl, pH 6.8 and centrifuged at 20,000× g for 45 min at 4°C. The resulting pellet was stored at −80°C until use. rOMPA was produced from *E. coli* as described previously by our group [40].

### Fibronectin-binding assays

Fibronectin-binding assays were performed as described previously [23], with some modifications. Briefly, *A. baumannii* ATCC 19606, 77 or 113-16 strain grown overnight at 37°C in MHB were resuspended in PBS and collected by centrifugation at 5,000× g for 10 min. Bacteria were washed twice in sterile PBS and resuspended in the same sterile buffer. Two assays were performed: i) 50 µL of bacterial suspension were mixed with 50 µL of PBS, added to BSA or fibronectin coated wells and incubated 3 h at room temperature, and ii) 50 µL of bacterial suspension were mixed with increasing amounts of human plasmatic fibronectin (0, 10, 100 and 1,000 µg/mL), added to fibronectin coated wells and incubated 3 h at room temperature for bacterial adsorption. After rinsing thrice with sterile PBS, 50 µL of bacterial suspension were added for 3 h at room temperature for bacterial adsorption. Non-adhered bacteria were discarded and wells were washed six times with sterile PBS to remove unbound bacteria. Adherent bacteria were then collected with 125 µL of sterile PBS containing 0.5% Triton X-100. Diluted lysates were plated onto sheep blood agar (Becton Dickinson Microbiology Systems, USA) and incubated at 37°C for 24 h for enumeration of developed colonies and then the determination of the number of bacteria that attached to fibronectin.

### Sodium dodecyl sulfate polyacrylamide gel electrophoresis (SDS-PAGE) and immunoblotting assay

After the determination of the OMPs and rOMPA amounts by using the bicinchoninic assay assay (Thermo Scientific, Spain), three or six µg of proteins were mixed with an equal volume of 2× Laemmli buffer, denaturated by heating the mixture for 5 min at 95°C, and then resolved by 10% SDS-PAGE. The gels were stained with SimplyBlue™ SafeStain following the manufacturer's instructions (Invitrogen, Spain).

### Western blotting

To investigate the possible involvement of OMPs and OMPA in the *A. baumannii* interactions with fibronectin, OMPs extracts and rOMPA were used for western blotting. Western blotting analysis was performed following the SDS-PAGE as indicated above. The separated proteins were transferred using nitrocellulose membranes (Amersham Bioscience, Spain), and the membranes were blocked for 1 h with PBS and 0.1% (v/v) Tween 20 (PBST buffer) containing 5% (w/v) milk. The nitrocellulose membranes were then incubated with human fibronectin (10 µg/mL) for 1 h, washed twice with PBST buffer, and incubated overnight at 4°C with primary antibody: rabbit anti-human fibronectin (1∶2,500 dilution) diluted in PBST buffer containing 5% milk. After washing with PBST buffer, the membranes were incubated for 1 h at room temperature with a horseradish peroxidase-conjugated goat anti-rabbit IgG antibody (Amersham Bioscience, Spain) (dilution 1∶10,000) diluted in PBST buffer containing 5% milk. Subsequently, immunoreactive proteins were visualized using the enhanced chemiluminescence protocol (Super ECL, (Amersham Bioscience, Spain).

For peptide mass fingerprinting, SimplyBlue™ SafeStain-stained bands representing FnBPs were excised from SDS-PAGE gel and sent to the Centro de Investigación Príncipe Felipe de Valencia for MALDI-TOF-TOF (MS-MS/MS) analysis. Data obtained from peptide mass fingerprinting were matched against the NCBI database (http://www.ncbi.nlm.nih.gov) using the Mascot program.

### Immunofluorescence

A549 cells plated on coverslips in 24 well plates for 24 h and infected with *A. baumannii* ATCC 19606, 77 or 113-16 strain at 37°C for 2 and 24 h were removed and washed five times with cold PBS. A549 cells were fixed in methanol for 8 min at −20°C, permeabilized with 0.5% Triton X-100 and blocked with 20% porc serum in PBS. Primary antibodies: mouse anti-*A. baumannii* OMPs [41] and rabbit anti-human fibronectin (Sigma, Spain) were used at dilution of 1∶50 and 1∶400 in PBS containing 1% BSA for 2 h, respectively. After washing with PBS, the coverslips were incubated with the secondary antibodies: Alexa488-conjugated goat anti-mouse IgG, and Alexa594-conjugated goat anti-rabbit IgG (Invitrogen, Spain) at dilution of 1∶100 and 1∶800 in PBS containing 1% BSA for 1 h, respectively. The fixed coverslips were incubated for 10 min at room temperature with DAPI (Applichem, Germany) (0.5 µg/mL), washed with PBS, mounted in fluorescence mounting medium (Dako Cytomation, Spain), and visualized using a Leica fluorescence microscope (DM-6000; Leica Microsystems Wetzlar GmbH, Germany).

### Adhesion assay

i) A549 cells were pretreated with RGD (0.5 and 1 mg/mL), a fibronectin inhibitor, for 30 min, rabbit anti-human fibronectin (28 µg/mL) for 60 min, mouse IgG (1∶25) for 60 min, or with rat anti-human E-cadherin (1∶25) for 60 min and infected with 10^8^ cfu/mL of *A. baumannii* ATCC 19606, 77 or 113-16 strain for 2 h with 5% CO_2_ at 37°C. ii) A549 cells were infected for 2 h in 5% CO_2_ at 37°C with 10^8^ cfu/mL of *A. baumannii* ATCC 19606, 77 or 113-16 strain previously incubated for 1 h at room temperature with mouse anti- *A. baumannii* OMPA (diluted in PBS at 1∶1,000 and 1∶250), mouse IgG (1∶25) for 60 min, or with rabbit anti-*A. baumannii* CarO (1∶25) kindly gift by AM Viale [42]. Subsequently, infected A549 cells were washed five times with prewarmed PBS and lysed with 0.5% Triton X-100. Diluted lysates were plated onto blood agar (Blood-Agar Columbia, Becton Dickinson Microbiology Systems, USA) and incubated at 37°C for 24 h for enumeration of developed colonies and then the determination of the number of bacteria that attached to A549 cells.

### Statistical analysis

Group data are presented as mean ± SEM. Student *t*-test was used to determine differences between means. Difference was considered significant at *P*<0.05. SPSS (version 15.0) statistical package was used (SPSS Inc., Chicago, IL).

## Supporting Information

Data S1 **TonB-dependent copper receptor sequence information.**
(DOC)

Data S2 **OMPA sequence information.**
(DOC)

Data S3 **34 kDa OMP sequence information.**
(DOC)
