# Supplementary material for: Role of Fibronectin in the Adhesion of Acinetobacter baumannii to Host Cells
Source: PLoS One. 2012 Apr 13;7(4):e33073. doi: 10.1371/journal.pone.0033073 (PMC3326023; doi:10.1371/journal.pone.0033073)
Supplement: Data S1 — TonB-dependent copper receptor sequence information. (DOC) [file pone.0033073.s001.doc]

**Supplemental data**

**Data S1. TonB-dependent copper receptor sequence information.**

**Band** 78.03 kda

**Sequence coverage (%)** 48

**Peptide matched**

1 MPHSKFLLQP LWVAMLAVSH SGLVFAESEK NDAETNTLHS LAPIVVTAQQ

51 GNDANGLIVH ADPKQPIQPV PATDGADYLQ SIMGFNSIQS GGTNGDVTFR

101 GMFGSRIKIL TDGTENLGAC PNRMDAPTSY ISPESYDRIS VIKGPQTVQY

151 ANTGSAATVL FERQPEKLTS EKPYRGQASV LLGSYGRIDH NVEAAIGDEK

201 KYIRLNANRS ESNSYQDGDG NTVPSAWKKW NADVALGFTP DENTWVEITG

251 GKSDGESLYA GRSMDGSQFA RESLGLRFEK KNITDVIKKI EGQVNYSYND

301 HIMDNFSLRT PPLVEMNHGG MTMLMPNAMA MQVTRRTLNS RLAMTSEWNK

351 WSLTTGVDSQ FNKHGGSMSS PTMPSMNVPY RQDMRFQSYG AFGELGYQWN

401 DQNKLVTGAR LDRVTVEDER TDSQAKGFNT KLEKTLPSAF VRWENQHPEH

451 DLKSYIGLGY VERMPDYWEL FSPKHGNAGS TNTFNGVNPE KTLQLDLGFQ

501 QQHGALNTWA SAYAGLVDDY ILMSYHHHPS MGMDGHDMSH DITAGAKNVD

551 ATIAGAEAGI GYQFTDRIQA DLSAMYAWGK NTTDDKPLPQ ISPLEGRLNI

601 RYVADKYNLG LLWRAVAEQK RVSLHQGNIV GYDLKPSKGF STLSLNGSYN

651 LRKDIDVSVG IDNVLDKTYT EHLNKAGSAG FGFASEEQFN NIGRNYWVRM

701 SMKF

**ID** gi:260557574

**Protein** TonB-dependent copper receptor
