# Supplementary material for: Role of Fibronectin in the Adhesion of Acinetobacter baumannii to Host Cells
Source: PLoS One. 2012 Apr 13;7(4):e33073. doi: 10.1371/journal.pone.0033073 (PMC3326023; doi:10.1371/journal.pone.0033073)
Supplement: Data S2 — OMPA sequence information. (DOC) [file pone.0033073.s002.doc]

**Supplemental data**

**Data S2. OMPA sequence information.**

**Band** 36.94 kda

**Sequence coverage (%)** 51

**Peptide matched**

1 MKLSRIALAT MLVAAPLAAA NAGVTVTPLL LGYTFQDSQH NNGGKDGNLT

51 NSPELQDDLF VGAALGIELT PWLGFEAEYN QVKGDVDGAS AGAEYKQKQI

101 NGNFYVTSDL ITKNYDSKIK PYVLLGAGHY KYDFDGVNRG TRGTSEEGTL

151 GNAGVGAFWR LNDALSLRTE ARATYNADEE FWNYTALAGL NVVLGGHLKP

201 AAPVVEVAPV EPTPVAPQPQ ELTEDLNMEL RVFFDTNKSN IKDQYKPEIA

251 KVAEKLSEYP NATARIEGHT DNTGPRKLNE RLSLARANSV KSALVNEYNV

301 DASRLSTQGF AWDQPIADNK TKEGRAMNRR VFATITGSRT

**ID** gi:129307154

**Protein** outer membrane protein A
