# Supplementary material for: Role of Fibronectin in the Adhesion of Acinetobacter baumannii to Host Cells
Source: PLoS One. 2012 Apr 13;7(4):e33073. doi: 10.1371/journal.pone.0033073 (PMC3326023; doi:10.1371/journal.pone.0033073)
Supplement: Data S3 — 34 kDa OMP sequence information. (DOC) [file pone.0033073.s003.doc]

**Supplemental data**

**Data S3. 34 kDa OMP sequence information.**

**Band** 32.09 kda

**Sequence coverage (%)** 60

**Peptide matched**

1 MKKLGLATAV LLAMTGAHAY QFEVQGQSEY VDTTANDKNF TGDVAGTFYL

51 KNVDTAKGPL AEAAFLNQAS SVSLGYSYQQ YDQNNVNYHI GTYGVKGEAY

101 VPTPYLPVYA SATYNHTDVD GKNNFSKDDN GDRYALEVGA MLLPNFLMTV

151 GYTSVANQFA LDNFGIIGNG IYSAVNQTAA IQNDQDAVTA RAKYVGPIDG

201 TNMAIGFEAA GAFGQENQYG LKTDLYLTPK LSVGATFVGN DGEADIKGND

251 LGEFRQAWGG NVNYFITPAL AVGASYMKAD VKKSSYDTQT IGLNAKFRF

**ID** gi: 184159810

**Protein** 34 kda outer membrane protein [Acinetobacter ACICU]
